# Self-Assessed Quality of Life Is Differently Impacted Depending on Diagnostic Grouping in Otorhinolaryngology: An Observational Study

**DOI:** 10.3390/healthcare13172239

**Published:** 2025-09-08

**Authors:** Dragica Severinac, Ines Begović, Emili Dragaš, Goran Geber, Davor Vagić, Andro Košec

**Affiliations:** 1 Department of Otorhinolaryngology and Head and Neck Surgery, University Hospital Center Sestre Milosrdnice, Vinogradska Cesta 29, 10000 Zagreb, Croatia; dseverinac@gmail.com (D.S.); ines.begovic123@gmail.com (I.B.); goran.geber1@gmail.com (G.G.); davorvagic1@gmail.com (D.V.); andro.kosec@yahoo.com (A.K.); 2 School of Medicine, University of Zagreb, 10000 Zagreb, Croatia

**Keywords:** quality of life, otorhinolaryngology, self-assessment, patient satisfaction

## Abstract

**Background and Objectives**: Quality of life is significantly impacted by patients’ diagnosis perceptions, and self-assessment of quality of life is increasingly used in medicine. With this study, we aim to provide an overview of the differences in self-assessed quality of life among patients in different diagnostic categories in otorhinolaryngology, focusing on physical health, physical pain, and social and emotional well-being. We hypothesize that these differences are substantial and can further improve patient care. **Methods**: The research was carried out from 1 May to 30 June 2024 with 127 otorhinolaryngology patients scheduled for a follow-up appointment at an otorhinolaryngology clinic. The 36-Item Short Form Health Survey, adequately completed by 114 patients, was used to determine the differences in self-reported quality of life between patients with different otorhinolaryngological diagnoses, classified by the International Classification of Diseases. **Results**: The results showed significant differences in the self-assessed quality of life for patients with different otorhinolaryngology diagnoses. Patients with oncological diagnoses had lower scores for emotional (*p* ≤ 0.001) and social functioning (*p* ≤ 0.038) compared with patients with other diagnoses but scored similarly to patients with chronic inflammation of the nose and patients with impaired communication due to ear or voice diseases. **Conclusions**: Self-assessment of quality of life is significantly different across diagnostic categories in otorhinolaryngology. These findings underscore the necessity of tailoring healthcare communication strategies to the specific needs of individual patients, addressing not only their physical but also emotional well-being.

## 1. Introduction

Self-assessment of quality of life is increasingly used in medicine and otorhinolaryngology and has become an important indicator of treatment success [1,2]. Quality of life (QoL) is not easy to define as it encompasses various aspects of one’s life. The literature provides various definitions. The World Health Organization defines it as a patient’s perception of their position in life in the context of the culture and value systems in which they live and in relation to their goals, expectations, standards, and concerns [3]. Health-related QoL assesses the impact of an illness on the physical, emotional, and social well-being of patients [2]. It can also be defined as the possibility that after the treatment is completed, the patient lives physically, mentally, and socially as closely as possible to life before treatment [3]. Considering the subjective nature of QoL assessment, there are various questionnaires for its measurement globally, one of which is the SF-36 questionnaire used in this study. According to Ware, the author of the questionnaire, health is measured multidimensionally, and therefore, overall quality of life is evaluated through physical health, perceived physical pain, and an individual’s social and emotional state, additionally assessing their mental health [4]. Physical pain and a consequent decrease in physical health, measured in the questionnaire by their impact on performing daily activities, indicate their significant role in most aspects of an individual’s life and consequently the social and emotional well-being of patients, as a decrease in the performance of daily activities often has a detrimental effect on mental health, and vice versa [4].

This study aims to evaluate the self-assessed quality of life in groups of otorhinolaryngology (ENT) patients with various conditions and provide more comprehensive insight into their specific healthcare needs. Studies currently present in the literature investigated individual diagnoses and their impact on the QoL in patients, whereas the goal of our study is to provide a comprehensive overview of the QoL of patients in different diagnostic categories in otorhinolaryngology [5,6,7]. We hypothesize that there are significant differences in the self-assessed QoL of patients in different diagnostic categories in the field of otorhinolaryngology. Our hope is to draw attention to tailoring patient communication and treatment based on the current differences in QoL in the hopes of enhancing patient satisfaction and, consequently, their QoL.

The aim of this research is to determine the differences in the self-assessed quality of life among ENT patients in different diagnostic categories, specifically through indicators of physical health, experienced physical pain, and their social and emotional well-being.

## 2. Materials and Methods

**Figure 1:**
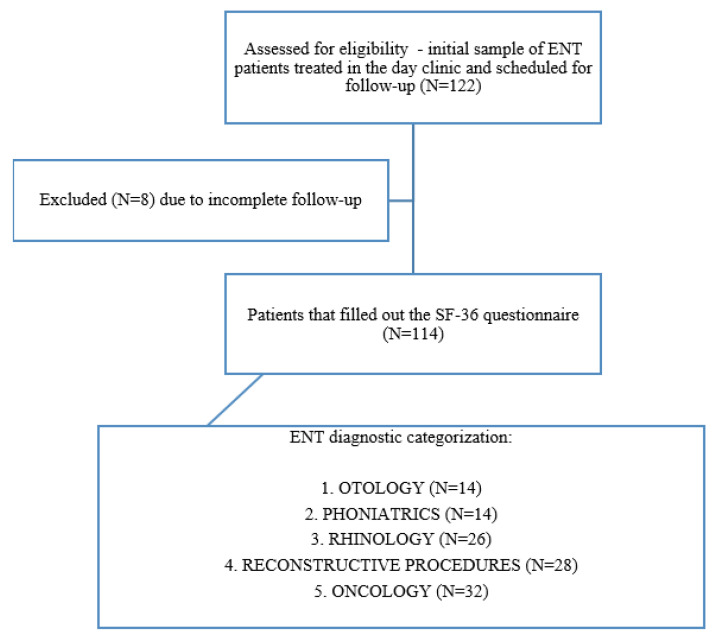
STROBE flowchart.

This observational study was conducted according to STROBE guidelines. The details of the patient selection process are provided in Figure 1.

A convenient sample of respondents was used. The research was an observational cross-sectional study aimed at assessing baseline QoL in different groups of ENT diagnoses prior to any surgical treatment carried out from 1 May to 30 June 2024. The study was designed to include patients coming to the ENT day clinic at the same time during the day who stayed in the same waiting space, were seen by the same ENT team, and were cared for by the same team of nurses and physician assistants. Every attempt was made to afford all respondents equal time to fill out the questionnaire. Inclusion criteria consisted of patients aged 18 and above that had a previously established ENT diagnosis, were scheduled for a follow-up examination at the day clinic, and had completed the survey, alongside providing informed consent. A total of 122 patients participated in this study. Exclusion criteria were failure to complete the survey and the inability to secure informed consent. In addition, patients with multiple diagnoses were excluded to minimize selection bias, as were patients that received any prior surgical treatment to minimize bias related to the effect of surgery or placebo-related effects. Overall, eight respondents were excluded from the study because they did not fill out the information about ENT diagnoses, and the final research sample consisted of 114 patients.

Upon arrival at the follow-up appointment, patients were informed that the research was being conducted, and by signing a written informed consent form, they voluntarily agreed to participate. The study was approved by the University Hospital Center Ethics Committee (No. 251-29-11-24-03).

According to the diagnosis group criterion, the patients were divided into 4 groups according to the current International Classification of Diseases (ICD):

1. OTOLOGY (diseases of the ear)

H60–H95 (diseases of the ear and mastoid processes).

The main grouping criterion was the fact that these diseases affect hearing and communication and are not characterized by pain or physical disability.

2. PHONIATRICS (voice disorders)

J38–J38.3 (diseases of the vocal cords);

R49 (voice disorders).

The main grouping criterion was the fact that these diseases affect voice quality and communication and are not characterized by pain or physical disability.

3. RHINOLOGY (nose and breathing diseases)

J30–J33.9 (diseases of the upper respiratory system, which include vasomotor, allergic, acute, and chronic diseases of the nose and sinuses with or without the presence of polyps).

The main grouping criterion was the presence of chronic nasal inflammation and allergies that primarily affect nasal breathing, chronic stress, and sleep quality and are not marked by significant pain or disability.

4. CONDITIONS THAT REQUIRE RECONSTRUCTIVE PROCEDURES

J34.2 (deviation of nasal septum and nasal pyramid);

J35 (chronic tonsillitis);

D21 (benign connective tissue neoplasms);

Q17.5 (congenital ear malformations: protruding ear);

H02.3 (blepharochalasia).

The main grouping criterion was the absence of any prior surgery or functional deficit and a primarily aesthetic concern in various ENT areas. This group was referred for a follow-up exam due to the absence of any inflammatory disease or functional difficulty, such as nasal tip or pyramid deformity in the J34.2 group or patients concerned with palatine tonsil size or halitosis in the J35 group. The study also included patients with protruding ears in the Q17.5 group and patients referred for possible upper eyelid reduction that did not impair vision to a significant extent in the H02.3 group.

5. ONCOLOGICAL DISEASES OF THE HEAD AND NECK

C00–C14 (malignant neoplasms of the lip, oral cavity and pharynx);

C30–C34 (malignant neoplasms in the respiratory system);

C73 (malignant neoplasm of the thyroid gland);

C43–C44.3 (malignant neoplasm of the skin);

D38.5 (neoplasm of uncertain or unknown behavior of other respiratory organs).

The main grouping criterion was a previously diagnosed malignant disease in the ENT area but prior to any surgery that might affect QoL. These patients were referred for a follow-up examination to discuss further treatment, revise existing recommendations, or schedule diagnostic procedures.

Our questionnaire consisted of two parts. The first part was anonymous and was used to gain insight into the sociodemographic characteristics of the patients. It contained seven questions about gender, age, marital status (unmarried, married, divorced, or widowed), professional qualification (primary education, secondary education, or postsecondary education), employment (employed, unemployed, student, or retired), ENT diagnosis, and proposed future treatment (conservative or operative). Respondents were instructed to circle the answers that applied to them or enter their ages and diagnoses. The second part consisted of the SF-36 questionnaire, a health status questionnaire consisting of 36 items. The test measures health multidimensionally through the prism of the following aspects: physical functioning, constraints in functioning due to physical health, physical pain, social functioning, impairments in physical functioning due to emotional difficulties, vitality, psychological health, and a general self-assessment of one’s health status. The Croatian licensed version of the Andrija Štampar School of Public Health at the University of Zagreb was used [8,9] (see Appendix A). The analysis of these aspects of one’s health and consequent QoL aims to analyze most of the contributors to a patient’s everyday functioning and consequent QoL with regard to the current diagnosis. It encompasses both physical constraints (physical pain, overall physical health status, and energy levels) and their role in everyday functioning, as well as the mental state and social functioning of patients, which have a reciprocal relationship [4].

The goal of the data analysis was to investigate the relationship between the subjective quality of life of patients and their specific otorhinolaryngological diagnoses, coded by the current International Classification of Diseases (ICD). The numerical values of all eight subcategories of the questionnaire were compared—physical functioning, physical pain, general health, vitality, social functioning, emotional state, and mental health—for patients within different groups.

Different areas of health were covered by a different number of questions, and their number was determined according to the psychometric criteria of reliability and validity. The question related to a change in health was shown separately. Each answer to the question was scored according to established norms. Therefore, the number of points obtained for each question was coded into standard values on a unique scale from 0 to 100 points, where a higher score indicates better health. A power analysis was conducted based on the average value of the SF-36 score according to the threshold value of a difference in questionnaire results of 5 points. Alpha was set to 0.05, with the power of the test being 80%. The power analysis set the minimum required sample size for the establishment of statistical significance to 78 respondents. The analysis of the normality of data distribution was verified by the Smirnov–Kolmogorov test, and according to the obtained results, non-parametric tests and the corresponding display were used, along with the appropriate representation of continuous values (arithmetic mean, standard deviation, and median). The data was analyzed with the Kruskal–Wallis test, using an implemented Dunn’s pairwise post hoc test option to correct for multiple analyses, and the Spearman rho correlation test. They were all two-way tests. Values of *p* ≤ 0.05 were considered statistically significant. Statistical processing and data analysis were performed using the MedCalc program (Version 11.2.1 © 1993–2010 MedCalc Software bvba Software, Broekstraat 52, 9030 Mariakerke, Belgium) and the SPSS program (Version 22.0. Released 2013. IBM SPSS Statistics for Windows, IBM Corp, Armonk, NY, USA).

## 3. Results

In order to obtain the best possible insight into the profile of the respondents who participated in the research, a comparison was performed among the group of diagnoses in which the respondents were classified and their sociodemographic data. Analysis of the demographic variables using Spearman’s rho correlation coefficient concluded that there were no significant correlations between the sociodemographic variables and the quality of life of the respondents (Table 1 and Table S1)

**Figure 2:**
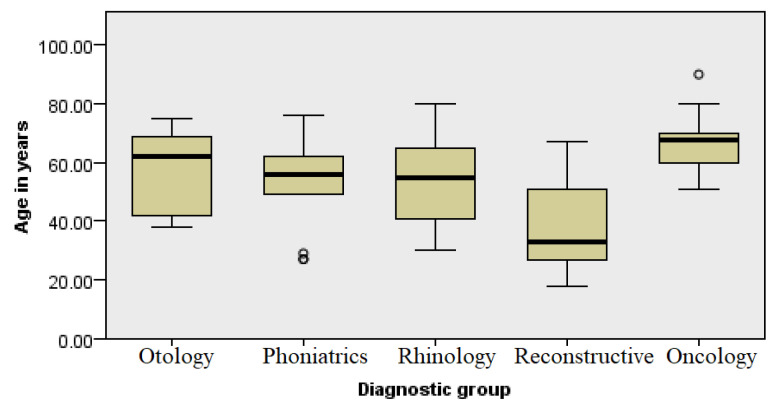
Analysis of patient age by diagnosis group.

Furthermore, the research analyzed the relationship between the age of the respondents and one of the four ICD diagnosis groups they were classified in (Figure 2). There were 14 respondents in group 1 (ear and hearing) (M = 7; F = 7). The distribution of respondents was equal according to gender, and the age in the group ranged from 27 to 76, with an average age of 53, while in group number 2 (voice diseases) (M = 7; F = 7), the average age was 49, ranging from 30 to 62. The total number of respondents in group 3 (diseases of the nose and paranasal sinuses) was 26 (M = 8; F = 18), aged from 30 to 80 years old, with an average age of 50 years. Group 4, which encompassed diseases that require reconstructive procedures, included a total of 28 (M = 10; F = 18) respondents, aged from 18 to 67 years old, with an average age of 38 years. The only group where the ratio was in favor of male patients was group 5 (oncological diseases of the head and neck), where out of a total of 32 patients, 22 were male, and 10 were female. Patients were aged from 19.5 to 90 years, with an average age of 66 years.

The multidimensional concept of health and self-assessment of health of the respondents (SF-36 questionnaire for subjective assessment of health) was analyzed through eight subcategories using the Kruskal–Wallis test.

### 3.1. Physical Functioning

**Figure 3:**
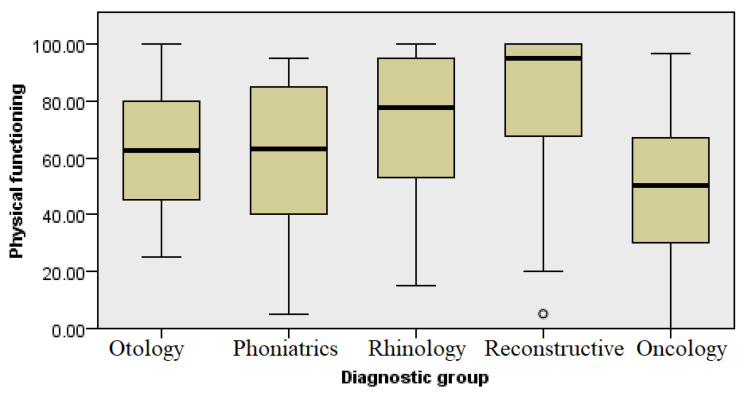
Analysis of physical functioning by diagnosis group.

A subcategory of the questionnaire refers to the activities that respondents engage in during a typical day. It includes 10 questions related to physically demanding activities (e.g., running or lifting heavy objects) and moderately strenuous activities (e.g., moving a table, playing bocce, cycling, lifting or carrying grocery bags, climbing stairs (several floors), climbing stairs (one floor), bending, kneeling, stooping, walking more than 1 km, walking 500 m, walking 100 m, bathing, or dressing) and how limited they are currently in the performance of these activities. This analysis showed variations in physical functioning (Figure 3) among different groups of diagnoses. Respondents from the five groups had a small but statistically significant difference in the self-assessment of physical functioning. The highest level of physical functioning, as expected, was experienced by the patients with aesthetic concerns (Kruskal–Wallis test, *p* ≤ 0.001).

### 3.2. Physical Health

**Figure 4:**
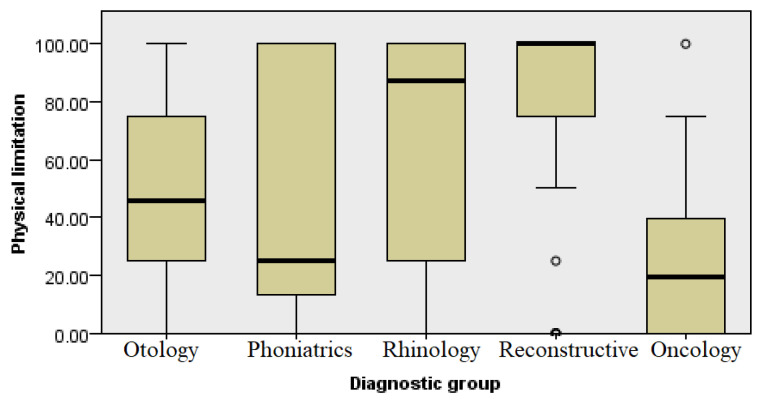
Analysis of physical health by diagnosis group.

The respondents answered four questions for a subcategory related to problems occurring in the previous month, such as reduced time spent at work or performing other activities, inability to perform a certain job or activity, and investment of additional effort to work or perform activities (Figure 4).

When analyzing the data on constraints related to physical health according to different groups, the oncological patients showed average results that were quite similar to the group of patients with ear, hearing, and voice diseases, while the rhinology patients and patients with aesthetic concerns had a significantly better self-assessment of physical constraints in relation to physical health (Kruskal–Wallis test, *p* ≤ 0.001).

### 3.3. Emotional Difficulties

**Figure 5:**
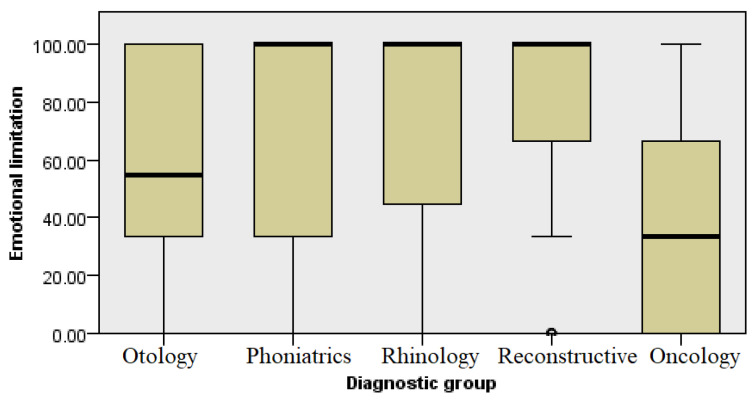
Analysis of emotional difficulties by diagnosis group.

Another subcategory compared the impact of emotional difficulties in the past month on the performance of work and daily activities. It contained three questions related to time spent working or carrying out activities, the amount of work being performed, and the maintained focus on work or activities performed by the respondents. The group of patients with aesthetic concerns had the highest level of emotional satisfaction, while the oncological patients had the lowest level of self-satisfaction (Figure 5) (Kruskal–Wallis, *p* ≤ 0.001).

### 3.4. Energy and Fatigue

**Figure 6:**
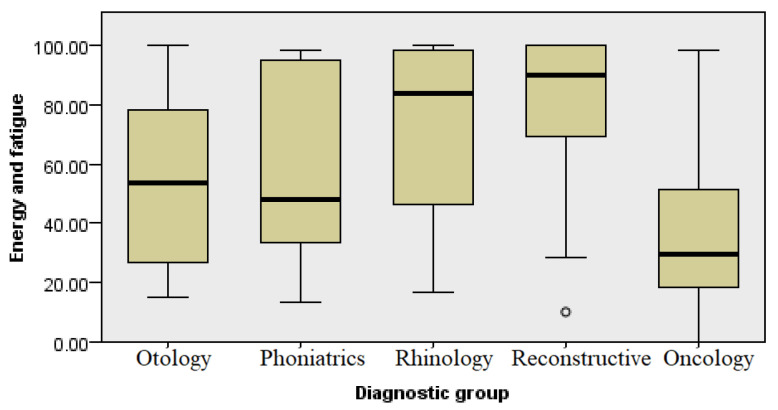
Analysis of energy levels and fatigue by diagnosis group.

This category contained four questions in which the respondents had to choose the answer that most accurately determined how full of energy, exhausted, or tired they felt. This analysis indicates statistically significant differences in the quantity of energy and fatigue (see Figure 6), where the oncology patients showed the lowest level of energy, followed by the otology patients. The respondents with aesthetic concerns, along with the rhinology patients, had high energy levels (Kruskal–Wallis, *p* ≤ 0.001) (Figure 6).

### 3.5. Emotional Well-Being

**Figure 7:**
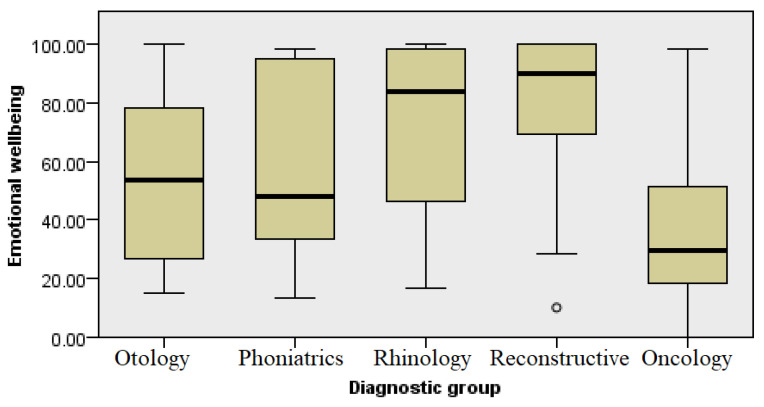
Analysis of emotional well-being by diagnosis group.

The data shown in Figure 7 shows a statistically significant difference in the results of the respondents in different groups in relation to emotional well-being. The respondents answered five questions related to their psychological states in the past month (how nervous, depressed, calm and peaceful, discouraged and sad, and happy they felt). The highest level of emotional well-being was expressed by subjects in the group with aesthetic concerns, followed by patients with diseases of the nose and paranasal sinuses with chronic inflammation, whereas a significantly lower level of well-being was reported by the respondents in the communication-impaired groups (1 and 2) alongside the oncology patients (Kruskal–Wallis, *p* ≤ 0.001).

### 3.6. Social Functioning

**Figure 8:**
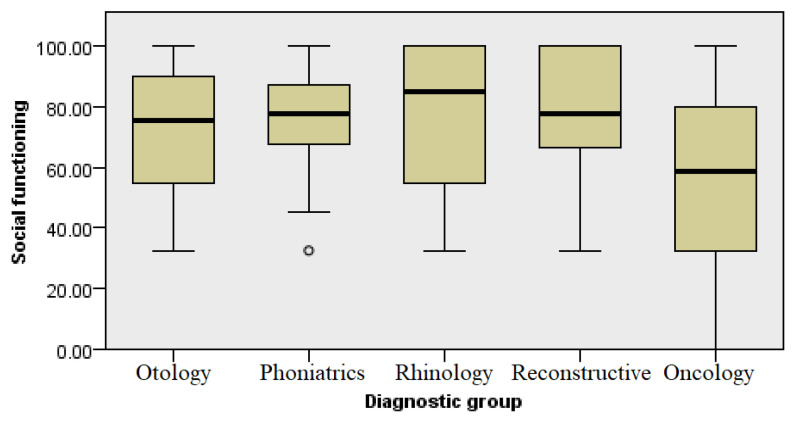
Analysis of social functioning by diagnosis group.

A subcategory of questionnaires included two questions assessing the extent to which physical health and emotional problems in the past month affected the usual social activities of the respondents with family and friends. The group of patients with oncological diseases of the head and neck had the lowest social level of functioning (Figure 8), although this level was surprisingly close to those of the other groups, whose levels of social functioning were slightly higher, but still statistically significant (Kruskal–Wallis, *p* ≤ 0.03) (Figure 8).

### 3.7. Pain

**Figure 9:**
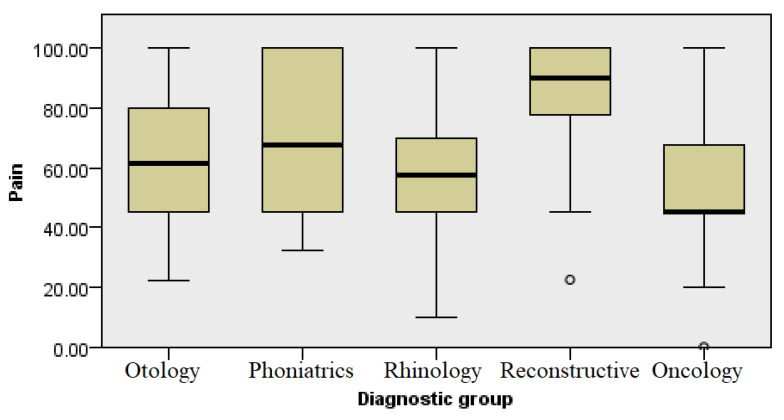
Analysis of pain by diagnosis group.

This category included two questions related to the amount of physical pain the respondents were under in the past month and to what extent the pain interfered with their routine occupational activities (including work outside of home and household activities). It is statistically significant that all groups were approximately the same regarding pain assessment, with a minimal difference present in the oncology patients compared with the communication-impaired and chronic inflammation groups. The subjects of the group that had aesthetic concerns expressed the lowest level of pain disturbance (Kruskal–Wallis, *p* ≤ 0.001) (Figure 9).

### 3.8. General Health

**Figure 10:**
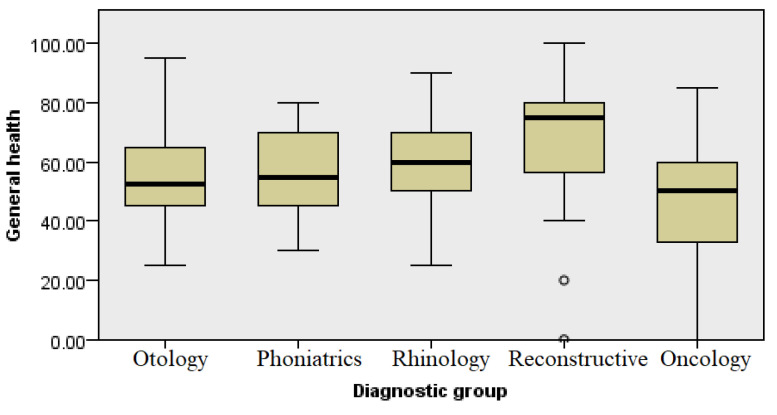
Analysis of general health by diagnosis group.

This category contained five questions related to general health. Respondents were asked to rate their current state of health and assess how their health compared to other people and whether they suspected it would deteriorate. The group undergoing reconstructive procedures showed the highest level of understanding of the concept of general health. The groups with diseases of the ear, hearing, and speech and oncology patients perceived the concept of general health almost identically (Kruskal–Wallis, *p* ≤ 0.004) (Figure 10).

## 4. Discussion

When interpreting the results of this research, it is important to emphasize that objective medical outcomes are important, but they are not the only accurate indicators of quality of life, as they significantly depend on the subjective perception of each patient.

There have not been many studies focused on comparing the perceived quality of life among otorhinolaryngological patients. Most of the studies currently present in the literature focus on one category of diagnosis, such as oncology, otology, or audiology, and specific diagnoses within these categories [5,6,7]. A study published in 2001 did compare the differences in QoL in otorhinolaryngology patients based on their diagnoses and demographic factors by using the SF-12 questionnaire [10]. Since the study was published over 20 years ago, and a more recent version of the questionnaire has since been published, we intended to fill this gap in the literature with our study.

By analyzing the sociodemographic data of respondents from different diagnosis groups, it is evident that younger women undergo plastic-reconstructive procedures more often, while men of older ages are diagnosed with head and neck cancers more often (Table S1 and Figure 2). The age difference should be considered when interpreting the results of the research, as some age and consequent comorbidities could have a role in the overall QoL of patients, especially considering the fact that the patients in the reconstructive group were significantly younger than the participants in the other groups. Other variables were not significantly associated with the self-assessed quality of life, which shows that the perceived quality of life is an inherent property independent of the level of education and marital status.

When assessing health status, the SF-36 questionnaire was used, which enabled a comprehensive assessment of the overall health status, with each diagnostic group showing a different average score in the eight questioned health domains. In the domain of physical functioning and health (Figure 3 and Figure 4, respectively), patients differed in their rehabilitation requirements and capacities for daily activities, with some requiring more extensive physical rehabilitation or additional support.

The emotional states and well-being of patients also differed by the different groups of diagnoses (Figure 5 and Figure 7, respectively). We could expect that this level would be significantly reduced in the oncology patients, primarily due to the social stigmatization associated with difficulties in communication, the need for frequent visits to the doctor as part of the long-term treatment, and the general challenges present in the treatment process of cancer patients. A decreased QoL has been noted in other studies, such as a study published by Berg et al. which demonstrated that patients with cancers at the base of the tongue have a lower QoL compared with the general population [5]. However, patients with ear, hearing, and voice diseases also showed low levels of emotional well-being (Figure 7). This can be associated with long-term treatment and restrictions that may affect daily activities, mostly related to personal hygiene and engaging in sports activities [11]. Difficulties in communication and acceptance of surdo-technical aids are still stigmatized in elderly patients, leading to isolation and thus impairment of their quality of life. Similar results were shown in a study published by Bakir et al., where patients with chronic otitis media reported significantly lower levels of QoL compared with a control group [6]. A decrease in QoL has also been noted in the literature among patients with functional voice disorders [7]. These results indicate that the pathologies classified within these groups of diagnoses can be considered significantly limiting diseases in otorhinolaryngology.

Low scores related to the self-assessment of energy and fatigue (Figure 6) in all four groups indicate a higher level of exhaustion due to underlying illness and the treatment process itself. The perception of pain in the group undergoing reconstructive procedures was at the lowest level and not significantly different when compared with other groups of respondents. This data can be used in healthcare planning and the application of continuous analgesia not only to oncology patients but also patients with other conditions and diagnoses in the field of otorhinolaryngology. Surgical resection followed by reconstruction in oncology patients may result in long-term pain, deformities, and possible limitations in shoulder and neck mobility. A loss of sense of taste often occurs after surgery which, in some patients, can be permanent, impairing the quality of life significantly more [12,13,14]. Alongside the use of continuous analgesia, emphasis should be put on the rehabilitation and treatment of patients [8]. The results of this research point to the need for individual medical care, whereby therapeutic approaches should be adapted not only to the patient’s physical condition but also the individual mental state, altering the guidelines and standard clinical practice to every individual whilst implementing shared decision making [15]. The introduction of psychosocial support can significantly improve the quality of life, especially for patients with functionally limiting diagnoses.

This research was limited to patients in the one department of ENT and head and neck Surgery, which may have affected the objectivity of results. The methodological approach is based on the subjective assessment and interpretation of patients, which can also affect the results obtained. QoL in general is affected by multiple and diverse variables, some of which are the economic state, support from the community, and the quality of treatment of each patient, all of which are variables which are hard to control and take into account. The reconstructive group had a significant age difference compared with the other four groups, while different possible comorbidities in oncologic patients could impact self-perceived QoL. Sociodemographic variables were tested and did not differ among the groups, but one questionnaire cannot assess the details of every diagnostic category in the same way. However, although flawed, the authors endeavored to ensure that the analysis was rigorous enough to draw attention to the results, indicating that health is affected by more than just the diagnostic category; it is also affected by the multidimensional aspects of disease and treatment in the ENT field.

## 5. Conclusions

This research confirms the hypothesis that there are significant differences in the self-assessment of quality of life among patients with different otorhinolaryngological diagnoses, but different diagnostic groups of patients also viewed themselves in a similar way where QoL was concerned. This underscores the importance of a personalized approach also prioritizing the patient’s emotional and social well-being, rather than solely focusing on the patient’s physical condition and diagnostic category.

## Figures and Tables

**Table 1:** The first part of the survey questionnaire.

Sex	Male	56 (47.1%)
	Female	63 (52.9%)
Age	Years of age	52.6 years;
		median = 57
Marital status	Unmarried	36 (30.3%)
	Married	64 (53.8%)
	Divorced	7 (5.9%)
	Widower or widow	12 (10.1%)
Level of education	Primary education (PE)	8 (6.7%)
	Secondary education (SE)	66 (55.5%)
	Postsecondary education (PSE)	45 (37.8%)
Employment status	Employed	53.8%
	Unemployed	5.9%
	Student	3.4%
	Retired	37%
Established Otorhinolaryngology Diagnosis		
Planned future treatment	Conservative	21.8%
	Operative	78.2%

**Figure A1:**
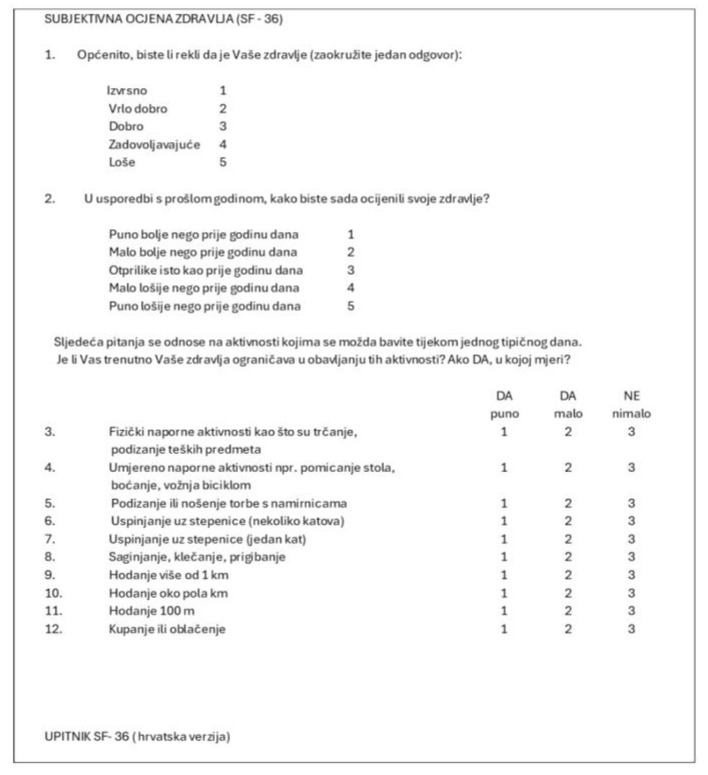
The first part of the Croatian licensed version of the SF-36 questionnaire by the Andrija Štampar School of Public Health.

**Figure A2:**
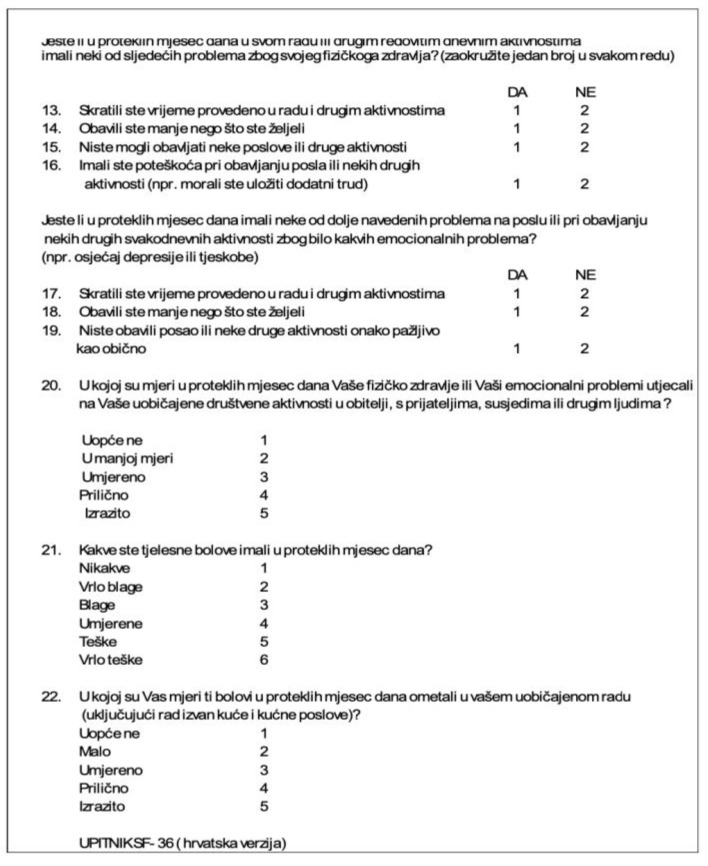
The second part of the Croatian licensed version of the SF-36 questionnaire by the Andrija Štampar School of Public Health.

**Figure A3:**
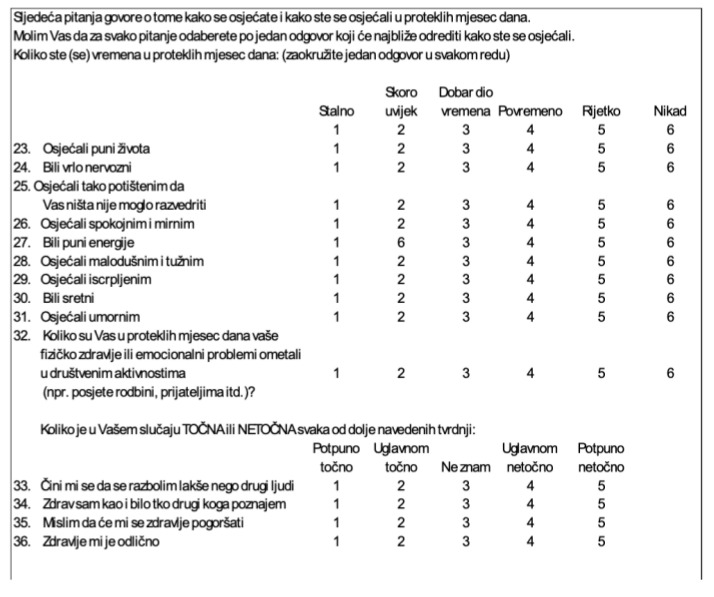
The third part of the Croatian licensed version of the SF-36 questionnaire by the Andrija Štampar School of Public Health [9].

**Figure A4:**
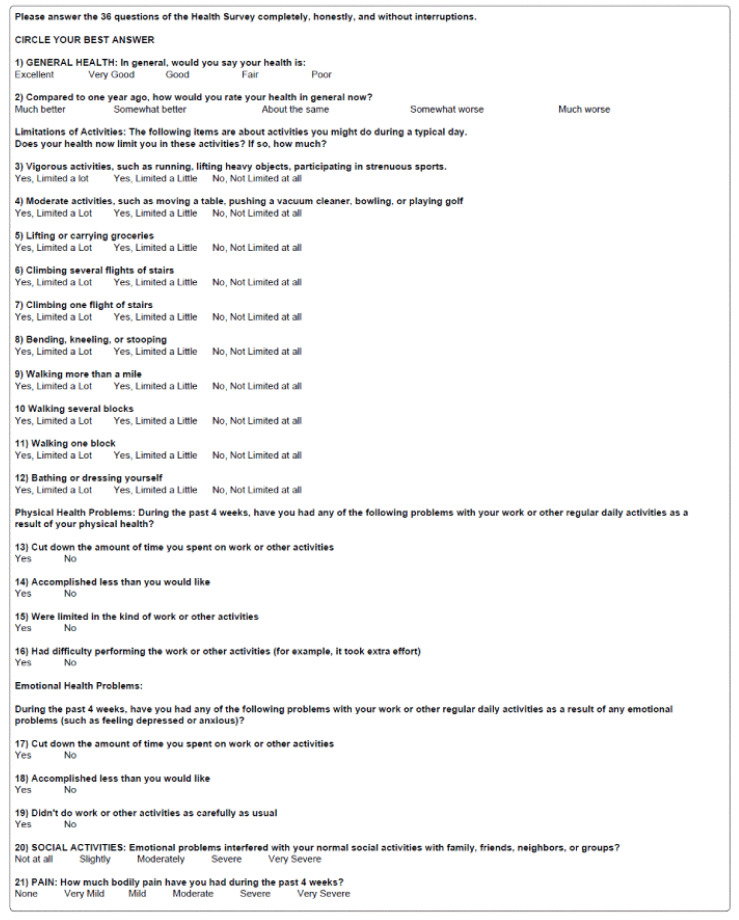
The first part of the English version of the SF-36 questionnaire [3].

**Figure A5:**
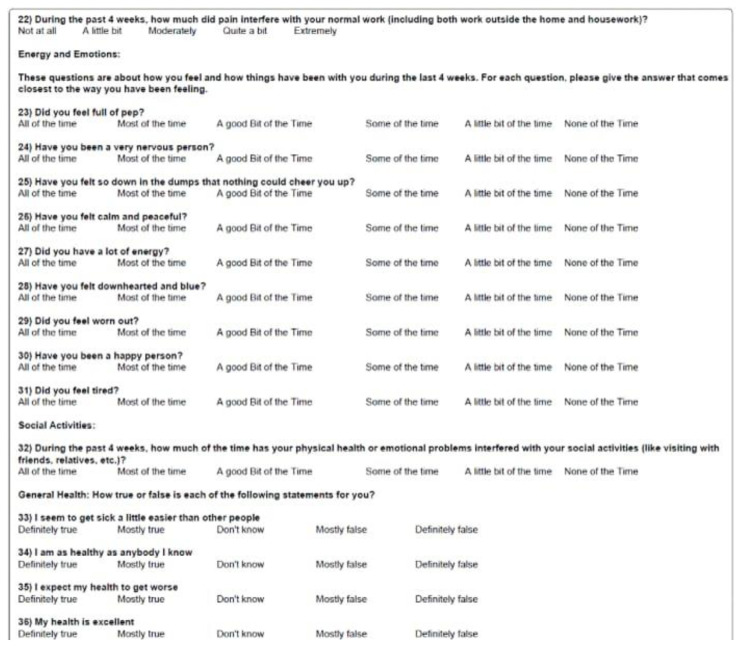
The second part of the English version of the SF-36 questionnaire [3].

## Data Availability

Data is available from the corresponding author upon reasonable request.

## Supplementary Materials

The following supporting information can be downloaded at https://www.mdpi.com/article/10.3390/healthcare13172239/s1. Table S1: Sperman’s rho correlation between the groups of diagnoses and the sociodemographic characteristics of the respondents.

## Abbreviations

The following abbreviations are used in this manuscript:

QoL	Quality of life
ENT	Otorhinolaryngology
ICD	International Classification of Diseases
PE	Primary education
SE	Secondary education
PSE	Postsecondary education

## References

[1] Vuletić G., Ivanković D., Davern M., Vuletić G. (2011). Kvaliteta života u zdravlju i bolesti. Kvaliteta života i zdravlje.

[2] Koenraads S.P., Aarts M.C., van der Veen E.L., Grolman W., Stegeman I. (2016). Quality of life questionnaires in otorhinolaryngology: A systematic overview. Clin. Otolaryngol..

[3] WHOQoL Group (1993). Study protocol for the World Health Organization project to develop a Quality of Life assessment instrument (WHOQOL). Qual. Life Res..

[4] Ware J.E. (2000). SF-36 health survey update. Spine.

[5] Berg M., Adnan A., Högmo A., Sjödin H., Gebre-Medhin M., Laurell G., Reizenstein J., Farnebo L., Norberg L.S., Notstam I. (2021). A national study of health-related quality of life in patients with cancer of the base of the tongue compared to the general population and to patients with tonsillar carcinoma. Head Neck.

[6] Bakir S., Kinis V., Bez Y., Gun R., Yorgancilar E., Ozbay M., Aguloglu B., Meric F. (2013). Mental health and quality of life in patients with chronic otitis media. Eur. Arch. Otorhinolaryngol..

[7] Andrea M., Andrea M., Figueira M.L. (2018). Self-perception of quality of life in patients with functional voice disorders: The effects of psychological and vocal acoustic variables. Eur. Arch. Otorhinolaryngol..

[8] Crnković I., Rukavina M., Ostrogonac K. (2015). Kvaliteta života laringektomiranih osoba. J. Appl. Health Sci..

[9] Maslić Sersić D., Vuletić G. (2006). Psychometric evaluation and establishing norms of Croatian SF-36 health survey: Framework for subjective health research. Croat. Med. J..

[10] Witsell D.L., Dolor R.J., Bolte J.M., Stinnett S.S. (2001). Exploring health-related quality of life in patients with diseases of the ear, nose, and throat: A multicenter observational study. Otolaryngol. Head Neck Surg..

[11] Schouwenaar E.M.M., Hellingman C.A., Waterval J.J. (2023). Health-related quality of life after otologic surgical treatment for chronic otitis media: Systematic review. Front. Neurol..

[12] Longacre M.L., Ridge J.A., Burtness B.A., Galloway T.J., Fang C.Y. (2012). Psychological functioning of caregivers for head and neck cancer patients. Oral Oncol..

[13] Vickery L.E., Latchford G., Hewison J., Bellew M., Feber T. (2003). The impact of head and neck cancer and facial disfigurement on the quality of life of patients and their partners. Head Neck.

[14] King S.N., Dunlap N.E., Tennant P.A., Pitts T. (2016). Pathophysiology of radiation-induced dysphagia in head and neck cancer. Dysphagia.

[15] Fenning S.J., Smith G., Calderwood C. (2019). Realistic Medicine: Changing culture and practice in the delivery of health and social care. Patient Educ. Couns..

