# Supplementary material for: Self-Assessed Quality of Life Is Differently Impacted Depending on Diagnostic Grouping in Otorhinolaryngology: An Observational Study
Source: Healthcare (Basel). 2025 Sep 8;13(17):2239. doi: 10.3390/healthcare13172239 (PMC12428553; doi:10.3390/healthcare13172239)
Supplement: Supplementary file 1 [file healthcare-13-02239-s001.zip › healthcare-3710225-supplementary.pdf]

Supplementary Files

Correlations									
			Diagnosis category	Sex	Age	Marital status	Level of education	Employment status	Treatment
Spearman's rho correlation	Diagnosis category	Correlation coefficient	1,000	-,154	,149	,029	-,102	,167	.336**
		Sig. (2-tailed)		,094	,107	,753	,270	,069	,000
		N	119	119	119	119	119	119	119
	Sex	Correlation coefficient	-,154	1,000	-,072	,067	,145	-,040	,031
		Sig. (2-tailed)	,094		,438	,471	,114	,665	,737
		N	119	119	119	119	119	119	119
	Age	Correlation coefficient	,149	-,072	1,000	.487**	-.323**	.736**	,084
		Sig. (2-tailed)	,107	,438		,000	,000	,000	,365
		N	119	119	119	119	119	119	119
	Marital status	Correlation coefficient	,029	,067	.487**	1,000	-,160	.223*	-,001
		Sig. (2-tailed)	,753	,471	,000		,083	,015	,992
		N	119	119	119	119	119	119	119
	Level of education	Correlation coefficient	-,102	,145	-.323**	-,160	1,000	-.351**	-,045
		Sig. (2-tailed)	,270	,114	,000	,083		,000	,630
		N	119	119	119	119	119	119	119
	Employment status	Correlation coefficient	,167	-,040	.736**	.223*	-.351**	1,000	,036
		Sig. (2-tailed)	,069	,665	,000	,015	,000		,701
		N	119	119	119	119	119	119	119
	Treatment	Correlation coefficient	.336**	,031	,084	-,001	-,045	,036	1,000
		Sig. (2-tailed)	,000	,737	,365	,992	,630	,701	
		N	119	119	119	119	119	119	119

*. Correlation is significant at the 0.05 level (2-tailed).

**. Correlation is significant at the 0.01 level (2-tailed).

Table S1. The correlation coefficient between the group of diagnoses in the ENT field and the sociodemographic characteristics of the respondents.
